# Quantitative Representativeness and Constituency of the Long-Term Agroecosystem Research Network and Analysis of Complementarity with Existing Ecological Networks

**DOI:** 10.1007/s00267-023-01834-9

**Published:** 2023-06-16

**Authors:** Jitendra Kumar, Alisa W. Coffin, Claire Baffaut, Guillermo E. Ponce-Campos, Lindsey Witthaus, William W. Hargrove

**Affiliations:** 1 Oak Ridge National Laboratory, Oak Ridge, TN USA; 2 USDA-ARS, Southeast Watershed Research Laboratory, Tifton, GA USA; 3 USDA-ARS, Cropping Systems and Water Quality Research Unit, Columbia, MO USA; 4 School of Natural Resources and the Environment, University of Arizona, Tucson, AZ USA; 5 USDA-ARS, National Sedimentation Laboratory, Oxford, MS USA; 6 USDA Forest Service, Southern Research Station, Asheville, NC USA

**Keywords:** Long-Term Agroecosystem Research Network - LTAR, Regionalization, Agroecoregion, Representativeness, Upscaling, Network design

## Abstract

Studies conducted at sites across ecological research networks usually strive to scale their results to larger areas, trying to reach conclusions that are valid throughout larger enclosing regions. Network representativeness and constituency can show how well conditions at sampling locations represent conditions also found elsewhere and can be used to help scale-up results over larger regions. Multivariate statistical methods have been used to design networks and select sites that optimize regional representation, thereby maximizing the value of datasets and research. However, in networks created from already established sites, an immediate challenge is to understand how well existing sites represent the range of environments in the whole area of interest. We performed an analysis to show how well sites in the USDA Long-Term Agroecosystem Research (LTAR) Network represent all agricultural working lands within the conterminous United States (CONUS). Our analysis of 18 LTAR sites, based on 15 climatic and edaphic characteristics, produced maps of representativeness and constituency. Representativeness of the LTAR sites was quantified through an exhaustive pairwise Euclidean distance calculation in multivariate space, between the locations of experiments within each LTAR site and every 1 km cell across the CONUS. Network representativeness is from the perspective of all CONUS locations, but we also considered the perspective from each LTAR site. For every LTAR site, we identified the region that is best represented by that particular site—its constituency—as the set of 1 km grid locations best represented by the environmental drivers at that particular LTAR site. Representativeness shows how well the combination of characteristics at each CONUS location was represented by the LTAR sites’ environments, while constituency shows which LTAR site was the closest match for each location. LTAR representativeness was good across most of the CONUS. Representativeness for croplands was higher than for grazinglands, probably because croplands have more specific environmental criteria. Constituencies resemble ecoregions but have their environmental conditions “centered” on those at particular existing LTAR sites. Constituency of LTAR sites can be used to prioritize the locations of experimental research at or even within particular sites, or to identify the extents that can likely be included when generalizing knowledge across larger regions of the CONUS. Sites with a large constituency have generalist environments, while those with smaller constituency areas have more specialized environmental combinations. These “specialist” sites are the best representatives for smaller, more unusual areas. The potential of sharing complementary sites from the Long-Term Ecological Research (LTER) Network and the National Ecological Observatory Network (NEON) to boost representativeness was also explored. LTAR network representativeness would benefit from borrowing several NEON sites and the Sevilleta LTER site. Later network additions must include such specialist sites that are targeted to represent unique missing environments. While this analysis exhaustively considered principal environmental characteristics related to production on working lands, we did not consider the focal agronomic systems under study, or their socio-economic context.

## Introduction

National and continental-scale ecological research networks are large, distributed, permanent investments, yet they provide a central organizational structure that permits coordinated observations over much larger extents than could otherwise be made by one or a few scientists. Network locations can be as elaborate as expensive infrastructure facilities, or as simple as locations where samples were grabbed. Regular, comparable, synoptic observations and measurements made over a large area can take advantage of natural experiments (Hargrove and Pickering 1992) and serendipity (Michener et al. 2009). However, thoughtful network design is necessary to ensure representation of diverse eco-climatological zones and broad application of network findings. The United States (US) National Science Foundation’s National Ecological Observatory Network (NEON) was one of the first networks to undergo a design phase before its construction, to optimize placement of observatory locations (Keller et al. 2008; Schimel et al. 2007). This planned approach provided a statistically valid methodology for intentional comparisons of ecological systems across regional domains, and for the exploration of variability within them (Keller et al. 2008).

Studies conducted across observational networks usually strive to scale up their results to even larger areas, trying to reach conclusions that are valid throughout regional, continental, and even global scales (Hargrove and Hoffman 2004a; Kitzes et al. 2021; Windsor et al. 2023). During initial network organization, however, factors like existing infrastructure, location of research institutions, and participation interest may take precedence over how well a location represents some, or all, of the larger area to be represented. The growth and addition of sites to such networks is often organic, resulting more from opportunity than from design. Initially, researchers rely on subjective expertise to judge how geographically far results obtained at one site might be validly extended. This process of network creation also poses an immediate problem for managers of the nascent network to determine how well the existing network of sites truly represents the entire area of interest which it claims. Network representativeness and constituency can show how well conditions at those locations represent conditions elsewhere within a larger area containing the network and can be used to help scale-up results over larger regions.

The availability of gridded geospatial data for environmental conditions, along with increased computing capacity, has allowed the use of quantitative multivariate statistical clustering methods to delineate geographic regions having similar conditions (Hargrove and Hoffman 2004b; Kumar et al. 2011; McMahon et al. 2004; White et al. 2005). Such clustering methods allow for the quantification of similarity, which can form the basis for an analysis of how well the environmental conditions at network sites represent the larger area which contains it (Hargrove and Hoffman 2004b; Hargrove et al. 2003). With these statistically based regionalizations, it is possible to know which site within a given network best represents any location in the area of interest, and how well it does so. With this knowledge, experimental results may thus be extrapolated to areas having quantitatively similar environmental conditions beyond the sites where they were obtained. Poorly represented areas in turn show the best candidates for new sites to be added to the existing network to maximize the representativeness of conditions within the greater area, thus providing directed network growth.

**Fig. 1:**
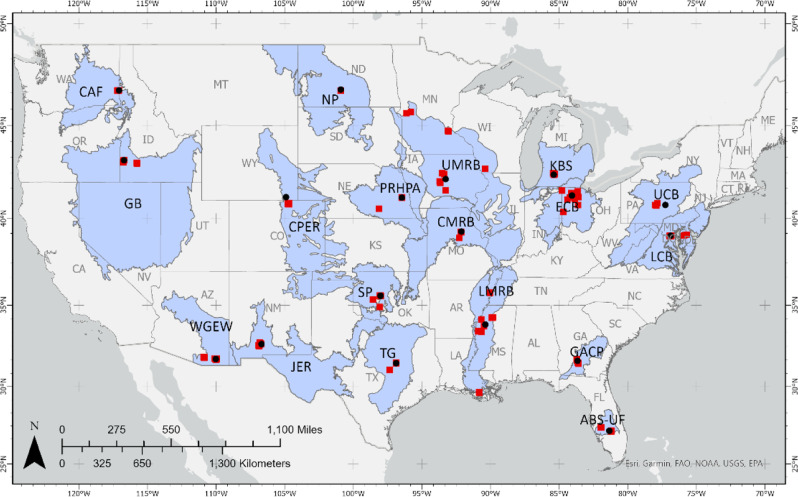
Locations of 18 Long-Term Agroecosystem Research (LTAR) Network sites across the conterminous US (CONUS). Black dots represent main site locations, red squares represent centroids of experimental plots or fields, and blue shaded areas represent locally estimated representative areas. (See Table 2 for LTAR site abbreviations; Armendariz et al. 2021; Bean et al. 2021; Coffin et al. 2020)

Agricultural land represented 44.4% of land cover in the US in 2020 (The World Bank 2023), with immense diversity in ecoregion characteristics and production activities across the country. There is a need to systematically study agroecological systems across the US to provide coordinated measurements and studies that represent the diversity of agricultural regions. To address this need, the Long-Term Agroecosystem Research (LTAR) Network was established by the US Department of Agriculture (USDA) in 2012 (Kleinman et al. 2018; Spiegal et al. 2018). Its objective is to carry out agricultural research that is long-term, transdisciplinary, and reaches across multiple sites and scales using a networked approach. The LTAR mission is to develop agricultural paradigms that are innovative and sustainable, based on evidence from regionally focused experiments. Research in the LTAR Network is coordinated among 18+ sites across the conterminous US (CONUS) (Fig. 1) that were selected, in part, to represent a diversity of agroecological systems in working lands. At a basic level, agroecological research in LTAR is grounded in the assessment of indicators of productivity, environment and well-being, a trio of domains rooted in early sustainability concepts described by the Brundtland Commission (World Commission on Environment and Development 1987). Using this general framework, LTAR plans to evaluate the effectiveness of solutions that compare the results of alternative production scenarios, modeled, and extrapolated across broad agricultural regions.

Research and data collection in LTAR is mostly organized around two main concepts: a common experimental framework, and long-term observatory sites on working lands. The common experiment seeks to compare Business-As-Usual (BAU) agricultural practices against those that are Aspirational (ASP). The practices explored in the common experiments are place-oriented, to reflect the agricultural economies and environments in their home region; however, these experiments are coordinated at the network level. In addition to the common experiment, LTAR also supports continued collection of data at long-term sites at USDA benchmark watersheds and experimental ranges. These include, for example, the Little River Experimental Watershed, Walnut Gulch Experimental Watershed, and the Jornada Experimental Range (Bosch et al. 2021; Goodrich et al. 2015; Havstad and Schlesinger 1996; Renard et al. 2008). A full description of each site’s research legacy is described in their initial proposals, available in the document archive on the LTAR website (https://ltar.ars.usda.gov/).

Although LTAR encompasses a variety of agricultural land-uses (i.e., rangeland, pastureland, and cropland), common measurements are collected at all sites to provide assessment of productivity (e.g., crop yields, forage yields, variables that quantify animal products), environment (e.g., variables that describe soil health, water quantity, water quality, air quality, and biodiversity), and well-being (e.g., farm income, costs of production, labor, profits). One key strength of the LTAR Network is the long-term collection of plot- and field-level management data that can be tied to these metrics. By identifying, measuring, and understanding these metrics and management data at LTAR sites, the LTAR Network is monitoring key indicators of sustainable production across working lands of the US (for example, Browning et al. 2021).

A central issue for LTAR is the challenge of extrapolating experimental results to broader regions. Accurately predicting broader landscape- to continental-level results of ASP treatments is key to effectively evaluating the tradeoffs among BAU and ASP scenarios and to understanding the potential effects of climate and other contextual changes. To accomplish this, it is necessary for researchers to understand the geographic representativeness of their respective LTAR site. Indeed, a regional representation of each LTAR site was included as one of the primary descriptors when each site entered the Network. The delineation of these representative regions was expert-based and derived by LTAR location scientists using non-standard processes. In 2018, these regional boundaries were codified and mapped, and in some cases adjusted, through an intensive, facilitated mapping exercise (Bean et al. 2021; Coffin et al. 2020). However, the regional boundaries defined in this exercise (Fig. 1, blue shaded areas) were still lacking in a standardized, quantifiable approach that could facilitate a scientific basis for extrapolating experimental results to broader areas. In addition, the regions fail to consider areas of the CONUS that fall outside of designated regions, with no information about how well these areas are (or are not) represented by existing LTAR sites. While the existing regional boundaries constitute a subjective expert-defined area that LTAR scientists can use to evaluate scenarios related to common LTAR experiments within their own regions, they are not effective in supporting wall-to-wall continental-scale models of agricultural outcomes in response to climate change.

The work presented here responds to two questions: how well does the LTAR Network represent agricultural production regions (i.e., working lands) of the CONUS, and its corollary, how well are working lands of the CONUS represented by the LTAR Network? Since LTAR experimental sites represent only a tiny fraction of the actual production landscape, and since the launching and management of a national scale network is such a vast and substantial undertaking, we also investigated the extent to which sites from the NEON and Long-Term Ecological Research (LTER) networks could possibly increase the overall LTAR representativeness using a strategy of cross-network site sharing. Through this potential “borrowing” step, we examined how LTAR could strategically partner with other national network sites to increase its representativeness of working lands.

## Methods and Materials

### Working Lands Masks

Rather than representing all lands, the goal of the LTAR Network is to specifically represent “working lands”. Essential systems of concern comprising working lands in the LTAR Network include croplands, grazinglands and “integrated systems”. Maps of all three of these types of working lands were developed as part of a tandem effort, which we used as masks, constraining our analysis of LTAR representativeness to areas where agricultural production was occurring or could potentially occur. Integrated systems are a combination of farm level cropping and animal systems and are areas where cropping and pasture or grazing systems exist in close proximity in space and time. Spatially defining integrated systems was complex and was dependent on knowledge of croplands and grazinglands. Therefore, the development of analytical masks focused on mapping croplands and grazinglands and relied upon the reclassification of multiple years of land cover data using the USDA Cropland Data Layer (Boryan et al. 2011) for the croplands mask, and the USGS Land Cover Database (Yang et al. 2018) for the grazinglands mask (Fig. 10). Croplands included lands dedicated to annual row crops, tree crops, hay, and silage. Potential grazinglands included grasslands, shrublands and emergent wetlands. Working lands originally developed at 30 m resolution were resampled at 1 km by identifying the dominant type within each 1 km pixel consistent with other datasets (Table 1) used in current analysis. These working lands masks were used to spatially constrain our quantitative representativeness analysis to show only areas in the CONUS where agriculture was occurring or was likely to occur.

**Table 1:** Gridded datasets used for the analysis and their sources

	Dataset title	Source
1	Annual mean temperature	WorldClim v2^a^
2	Isothermality	WorldClim v2^a^
3	Temperature seasonality	WorldClim v2^a^
4	Mean temperature of warmest quarter	WorldClim v2^a^
5	Mean temperature of coldest quarter	WorldClim v2^a^
6	Annual precipitation	WorldClim v2^a^
7	Precipitation seasonality	WorldClim v2^a^
8	Precipitation of warmest quarter	WorldClim v2^a^
9	Precipitation of coldest quarter	WorldClim v2^a^
10	Available water capacity	SoilGrids250m^b^
11	Soil bulk density	SoilGrids 2.0^c^
12	Soil carbon content	SoilGrids 2.0^c^
13	Soil nitrogen content	SoilGrids 2.0^c^
14	pH	SoilGrids 2.0^c^
15	Compound topographic index	HYDRO1K^d^

^a^ Fick and Hijmans (2017)

^b^Hengl et al. (2017)

^c^Poggio et al. (2021)

^d^Earth Resources Observation And Science (EROS) Center (2017)

### Datasets

A set of fifteen bioclimatic, edaphic, and topographic variables were selected for the analysis (Table 1). The variables were selected to capture and characterize the primary growing conditions, or environmental drivers, for working lands. Temperature and precipitation during the growing season are primary determinants of crop growth and yield and were captured by bioclimatic variables: *Annual Temperature*; *Mean Temperature of Warmest Quarter*; *Mean Temperature of Coldest Quarter*; *Annual Precipitation; Precipitation of Warmest Quarter;* and *Precipitation of Coldest Quarter*. Agricultural yields are also highly sensitive to inter- and intra-annual variability and fluctuations in climate, leading to extreme heat and drought conditions (Eck et al. 2020; Wolfe et al. 2018). Seasonal to diurnal variability in temperature conditions have been shown to have significant impact on crop yields (Lobell 2007; Xie et al. 2022). We included *Isothermality*, *Temperature Seasonality*, and *Precipitation Seasonality* variables to capture the variability and extremes in the growing conditions. All bioclimatic variables were derived from the WorldClim v2.1 database (Fick and Hijmans 2017). Soil properties and fertility are vital for crop growth and yield and were captured by the SoilGrid 2.0 datasets, including: *Available Water Capacity; Soil Bulk Density*; *Soil Carbon Content; Soil Nitrogen Content;* and *pH* (Batjes et al. 2020). The *Compound Topographic Index* was included to capture the topographic and soil wetness conditions (Earth Resources Observation And Science (EROS) Center 2017). All datasets were processed and harmonized to a 1 km x 1 km grid.

**Fig. 2:**
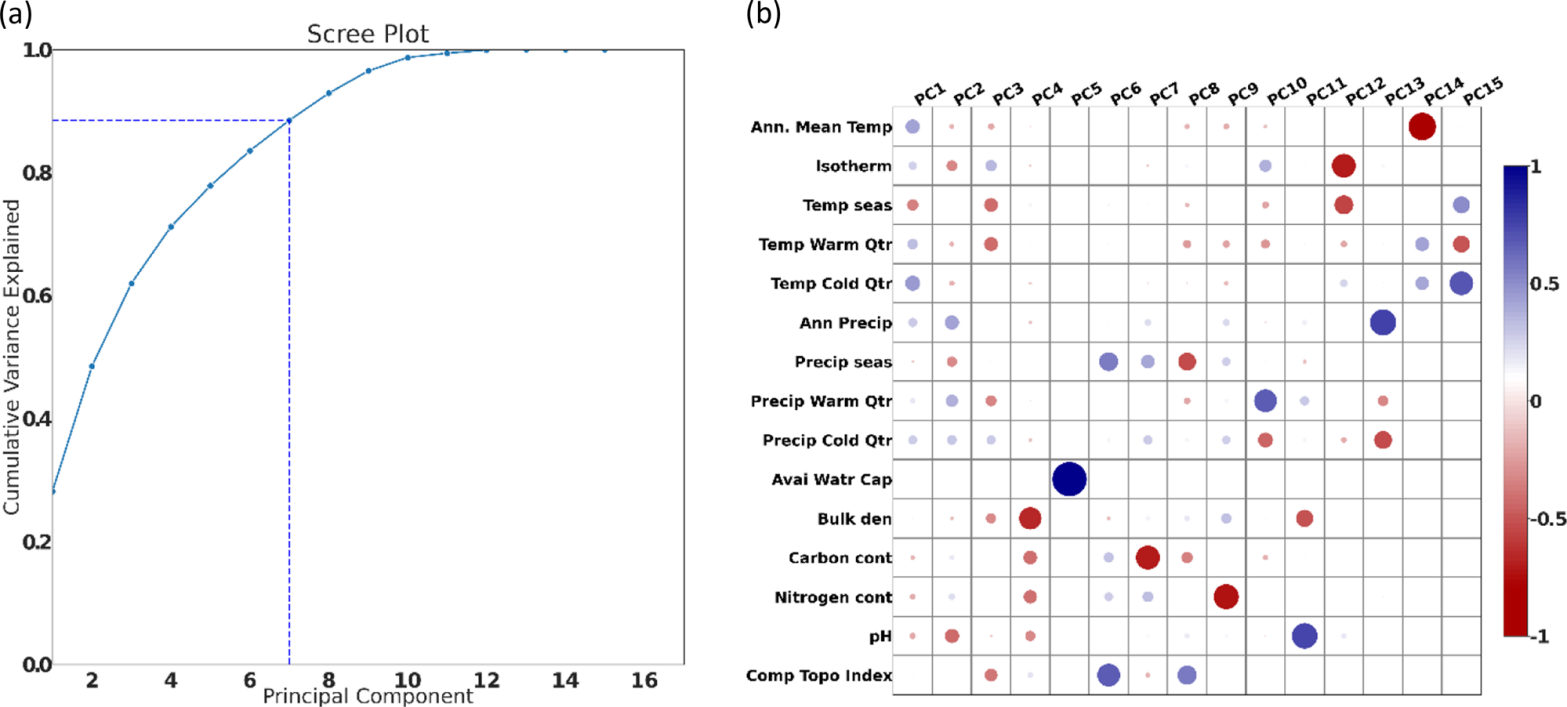
Principal component analysis of 15 selected environmental driver variables: **a** Scree plot showing cumulative variance explained by each component; **b** Principal component factor loadings

To prevent bias due to correlation among the selected set of variables, a Principal Component Analysis (PCA) was performed to extract features from the data into independent principal components. Principal components were calculated by performing a Singular Value Decomposition of the data using the PCA module within Python Scikit-learn (Pedregosa et al. 2011). After examining the eigenvalues (Fig. 2a), the top seven components explaining almost 90% of variance in the data were selected for analysis in this study. All results and analysis presented hereafter were conducted in this 7-dimensional Principal Component space.

The first principal component (PC1) explains 28% of the variance (Fig. 2a) and eigenvectors load primarily on temperature variables (Fig. 2b) emphasizing warm conditions year-round. The PC1 values represent year-round warm temperatures, and are high in the southern US, the arid southwest and the coastal region of the Pacific Northwest (Fig. 12a). Principal Component 2 (PC2) explains 20% of the variance (Fig. 2a) and loads positively on precipitation variables and negatively on temperature (Fig. 2b). The PC2 year-round precipitation values are low in the arid southwestern US, dominated by low height woody vegetation, while values are high in the wet forested ecosystems of the Pacific Northwest, northeastern, and eastern US (Fig. 12b). Principal Component 3 (PC3) explains 13% of the variance and loads positively on cold season precipitation, but negatively on warm season temperature and precipitation, soil bulk density, and compound topographic index. The PC3 precipitation as snowmelt values are low in the midwestern US, Mississippi River Basin, and higher in the Pacific Northwest. Principal Component 4 (PC4) explains 9% of the variance and loads negatively on soil properties, including bulk density and pH, representing soil consolidation and texture conditions. The PC4 values are low in the sandy coastal plain and the high mountain areas with uncompacted soils, higher in regions with more friable soils such as the midwestern US, and very high in urban areas with highly compacted soils. Principal Component 5 (PC5) explained 7% of the variance, and load primarily on soil available water holding capacity. The PC5 values are high in coastal Washington (WA), Oregon (OR), and California (CA), while low in the mountainous regions of Appalachia and large portions of Nevada (NV), Utah (UT), Colorado (CO) and Wyoming (WY). Principal Component 6 (PC6) explains 6% of the variance and loads positively on precipitation seasonality, soil carbon and nitrogen content, and compound topographic index. Principal Component 7 (PC7) explains 5% of the variance and loads positively on precipitation seasonality, negatively on soil carbon content, and positively on nitrogen content.

### LTAR Network Experimental Sites

**Fig. 3:**
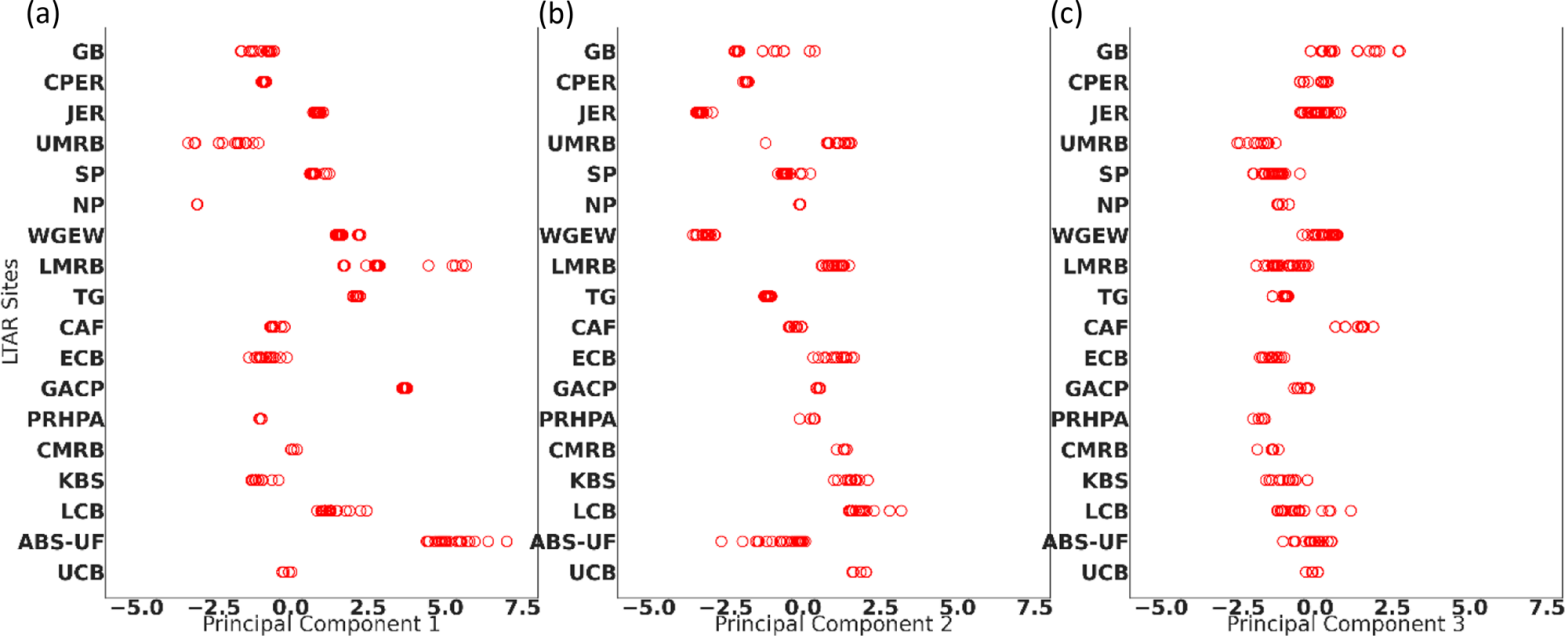
Plots of values for the first three principal components: **a** PC1 is interpreted as year-round warmth; **b** PC2 is year-round precipitation; and **c** PC3 is the ability to store winter precipitation in the soil. Experimental boundaries (red circles) within each site represent a range of variability in their local LTAR site conditions. Multiple centroids falling within the same 1 km cell will have identical environmental conditions

Each LTAR site consists of a set of geographically separate experimental boundaries that sample a range of environmental conditions at the site. These experimental site locations were taken from the LTAR Standard GIS Data Layers “Experimental Boundaries” vector polygon feature class (unmasked version, Armendariz et al. 2021) with polygon areas ranging from <1 ha plots to entire fields (>10 ha). To preserve the within-site environmental diversity, the centroid of each experimental polygon boundary was considered, creating a subnetwork of points for each LTAR site that was used for all calculations. A total of 1529 centroids in the 18 LTAR subnetworks (Table 2) were used to calculate the representativeness and constituency of each LTAR site by intersecting them with the CONUS 1 km grid data (Table 1) and extracting their environmental driver principal component values. Centroids falling in the same grid cell had identical environmental conditions. The LTAR sites varied widely in terms of the number and spatial distribution of centroids, making the sample size within each site different. Mean conditions were calculated as the mean across all centroids within a site’s subnetwork. Figure 3 shows the conditions sampled by every centroid within each LTAR site in the first three principal components space.

### Quantifying Network Representativeness and Site Constituency

Euclidean distance between two sites plotted in multivariate environmental space can be used as an inverse measure of multivariate similarity to quantify representativeness. Close sites in environmental space have a similar combination of environmental factors, and therefore are highly representative of each other. Multivariate clustering has also been widely used to segment the landscape, based on selected variables, to identify groups of map cells having similar environmental characteristics. Hargrove et al. (2003) used climate, soil, and topography to stratify the environments within the continental United States into a set of customized ecoregions using multivariate clustering, and then computed the representativeness of each resulting ecoregion to the most similar site within the existing AmeriFlux network. Sulkava et al. (2011) used a similar clustering approach using climate, soil, vegetation, and remote sensed data to quantify representativeness of the European flux tower network. Such an approach was successfully used to produce the environmental “domains” for the design of NEON (Keller et al. 2008; Schimel et al. 2007). Villarreal et al. (2018) used a maximum entropy approach to examine ecological functional type representativeness of Ameriflux and NEON flux tower locations and their core sites for the CONUS. Malone et al. (2022) applied a clustering-based approach to assess the representativeness of methane observing towers. Villarreal and Vargas (2021) used a random forest-based species distribution model approach to delineate the spatial distribution of environmental factors that are similar to the environmental range monitored by corresponding observation sites in Latin America. Most of these approaches can be broadly summarized as: 1) classification of multivariate space in clusters/strata having similar conditions; and 2) assessment of conditions captured by the observation sites within those clusters/strata.

Classifying the area intended to be represented in multivariate space provides generalized, data-defined clusters/strata, simplifies the computational problem, and is well-suited for the initial design of a new network, as was done for NEON. For existing observation networks, these methods offer a quantification of network representativeness relative to a theoretical baseline. These methods are advantageous for 1) evaluating clustering of variables for observational network design, and 2) for evaluating the representativeness of a particular observation relative to a broader network. However, the goal of the current study was to quantitatively assess the representativeness and constituency of the *already-existing* LTAR network of sites. As such, the environmental conditions at the existing LTAR sites already represent cluster centroids, even if non-optimal, of regions they are intended to represent. Hence, no clustering or grouping of environmental conditions was done here; instead, the combination of environmental drivers at every 1 km cell in the CONUS was exhaustively compared with each centroid from every LTAR site.

Representativeness of the LTAR sites was quantified through an exhaustive pairwise Euclidean distance calculation in multivariate space, between the experimental boundary centroids of each LTAR site (1529 centroids) and every 1 km cell ( >12 million) across the CONUS. Representativeness was calculated at each grid cell as a normalized index between 0 (least representative) and 1 (most representative) provided in Eq. 1, where *V*_*n*_ refers to the principal compoonents (*n* = 1 to 7) of variables in Table 1. The second term of Eq. 1 represents normalized distance (between 0 and 1) in 7-dimensional data space. This produced 1529 1 km gridded maps showing the environmental representativeness of each centroid at every location in the CONUS. Representativeness maps pertaining to the group of centroids comprising each of the LTAR sites were summed to produce a unique *site representativeness* layer for each LTAR site (i.e., one per LTAR site). The 1529 representativeness layers were then stacked, and by selecting the maximum value for each 1 km CONUS grid point in the layer stack, we produced the final map of LTAR *network representativeness*. Computation of representativeness and constituency was produced using a parallel analysis code developed with C programming language, run on a Linux-based high performance computing system. All statistical analysis were conducted using Python-based scripts (Kumar 2023).

1 $$representativeness = 1 - \left| {\sqrt {\mathop {\sum}\nolimits_{n = 1}^7 {\left( {V_n^{site} - V_n^{pixel}} \right)^2} } } \right|$$

Network representativeness is from the perspective of all CONUS locations, but we also considered the perspective from each LTAR site. For every LTAR site, we identified the region that is best represented by that particular site, referred to as the LTAR sites’ *constituency*, since these are the set of 1 km grid locations best represented by the environmental drivers at that particular LTAR site; all CONUS locations have a single LTAR site that best represents it. However, the richness of the data allowed for a deeper look into the representativeness of LTAR sites. Therefore, in addition to the most representative LTAR site, we computed a sorted list of the representativeness of all remaining LTAR sites for each 1 km CONUS grid location.

There was no enforcement of spatial contiguity; any spatial contiguity in the constituency map emerged due to the spatial autocorrelation of the environmental drivers across geographic space. Given the existing constellation of LTAR sites, the resulting constituency map represents the areas to which knowledge gleaned from each LTAR site about crop growth and production might be best generalized. The representativeness map shows the potential validity of such generalizations for each CONUS location, based on similarity with primary environmental drivers of production.

## Results

### Representativeness and Constituency of LTAR Sites

**Fig. 4:**
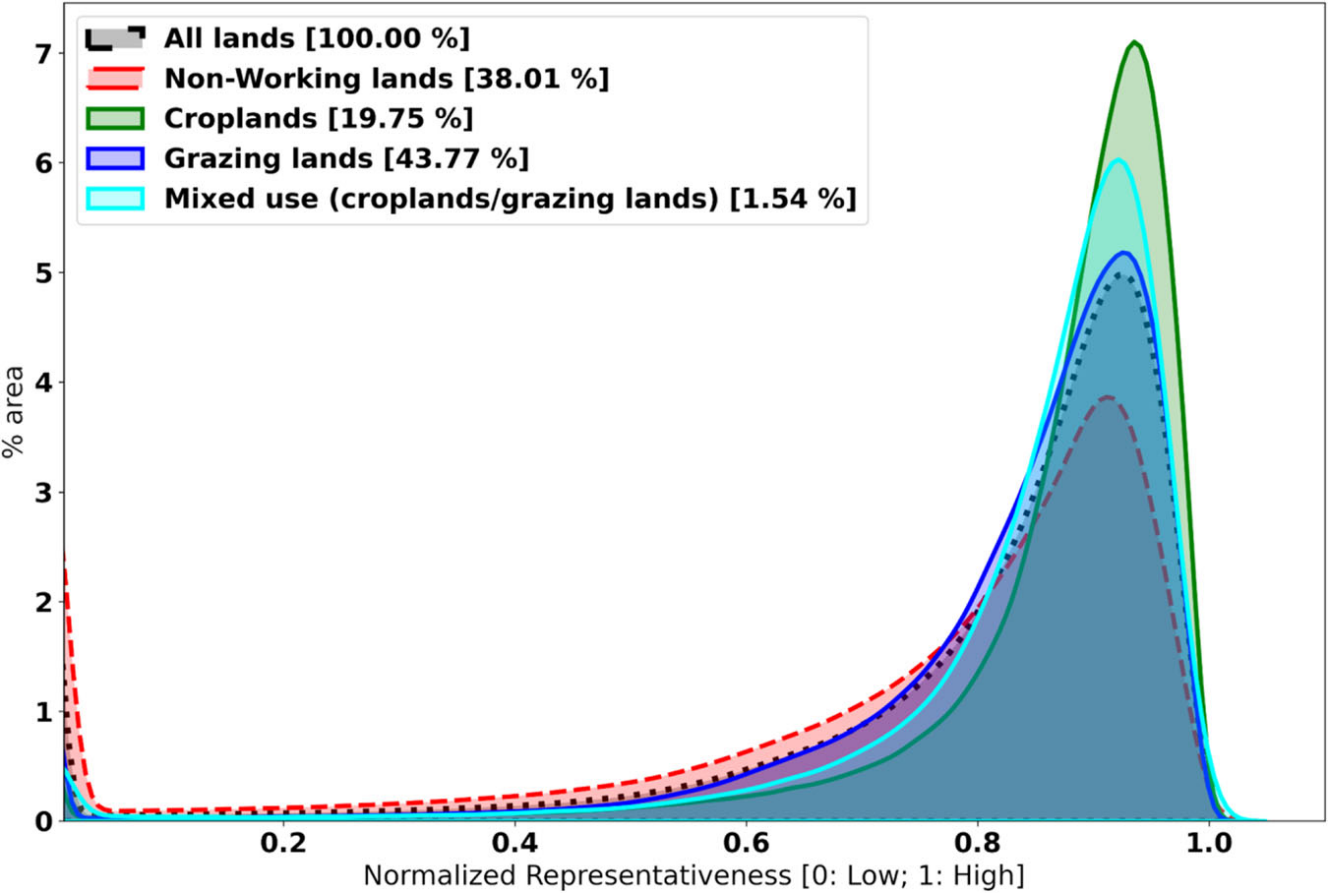
Distribution of normalized representativeness provided by the LTAR Network for various land cover types across the CONUS. The increased height of the peak indicates higher normalized representativeness. Croplands (green solid line) are best represented by the network, followed by mixed-use (croplands/grazinglands; cyan solid line), and grazinglands (dark blue solid line), with lowest representativeness for non-working lands (red dashed line)

The LTAR Network of 18 sites, with 1529 experimental boundary centroids, has good overall representativeness (0, not representative; 1 most representative) for all CONUS land cover types (mean: 0.78, median: 0.86) with a wide range of variability (standard deviations: 0.24) (Fig. 4). Croplands (~ 19.75% of land area) are best represented by the LTAR Network with mean representativeness of 0.86 (median: 0.90) and a standard deviation of 0.15. Grazinglands occupy a large fraction of the CONUS (~ 43.77% of land area), with a slightly lower mean representativeness of 0.81 (median: 0.87) and a higher standard deviation of 0.18. Land areas where croplands and grazinglands are highly integrated and mixed occupy a much smaller area (~ 1.54% of land area); and they have a mean representativeness of 0.83 (median: 0.88) and a standard deviation of 0.18. Non-working lands (~ 38% of land area), which are not targeted by the LTAR Network, are represented relatively poorly, with mean representativeness of 0.70 (median: 0.82) and a standard deviation of 0.30. Croplands require more specific environmental conditions, and so they may be easier to classify. Although representation of non-working lands is not a goal of the LTAR Network, the Network’s experimental areas nevertheless capture a fair representation of these non-target areas.

**Fig. 5:**
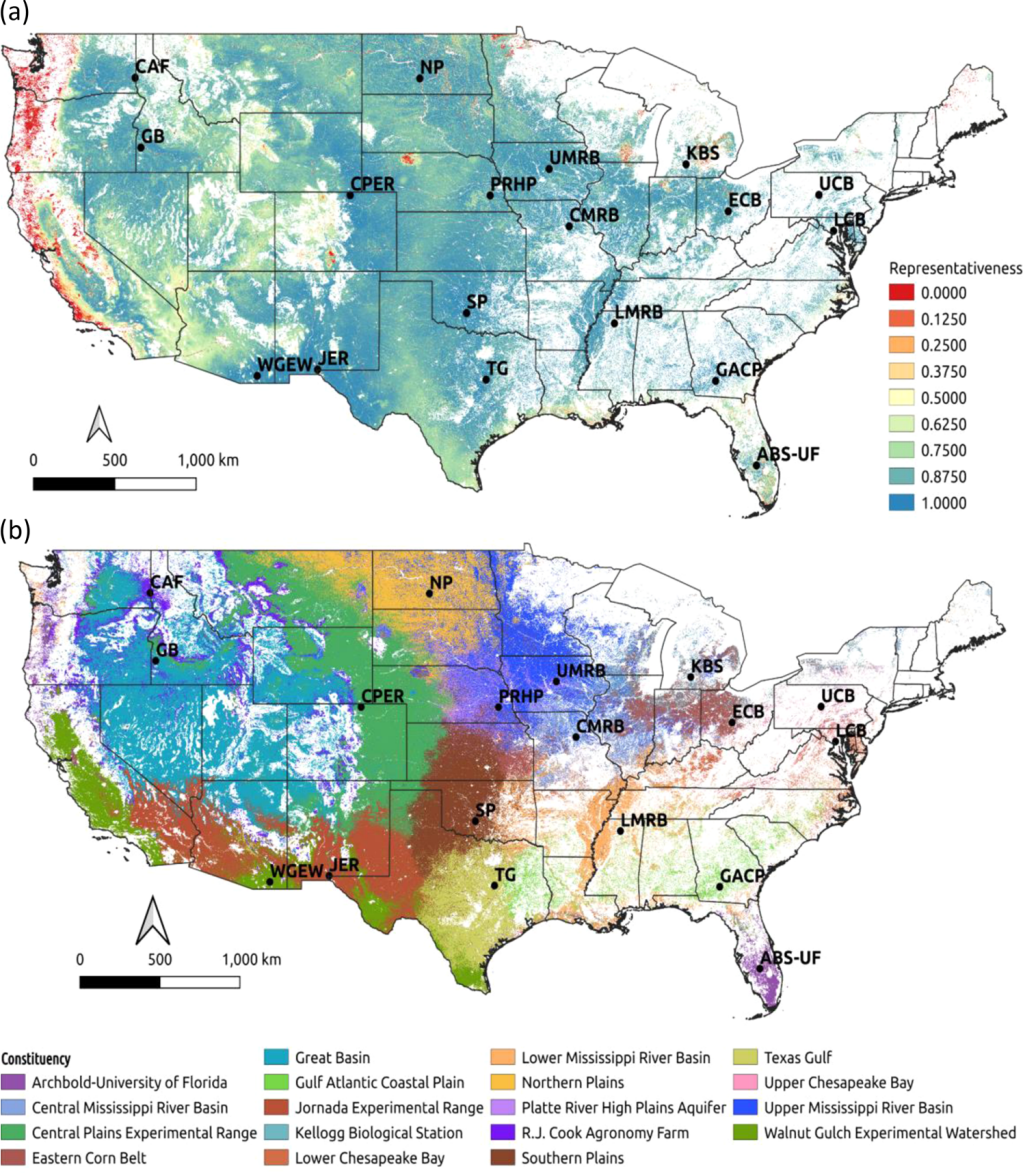
Representativeness and constituency maps of LTAR sites: **a** Normalized LTAR Network representativeness for all working lands across CONUS, showing least representative (0) to most representative (1); **b** Constituency of 18 LTAR sites across all the CONUS working lands. With only a few exceptions, most of the environmental driver conditions within the CONUS are well-represented by sites within the LTAR Network. The constituency map strongly resembles an ecoregion map, but each constituency region is anchored by the environmental conditions found at a particular LTAR site

Results of the analysis produced a map that describes the normalized representativeness of the LTAR Network (Fig. 5a) for CONUS working lands as defined by the cropland and grazinglands masks (Fig. 10a). Croplands primarily located in the eastern and midwestern CONUS are well represented by LTAR sites, with relatively low representativeness for more isolated croplands in northeast Michigan (MI) near Lake Huron, northern Vermont (VT) and Maine (ME). Although thirteen LTAR sites focus on the study of grazinglands, due to the vast extent and heterogeneity of this land use, their representativeness is highly varied. In the eastern United States, grazinglands interspersed between croplands are generally very well represented. Wetlands on the Atlantic and Gulf coastal margins, and the Greater Florida Everglades, considered to be potential grazinglands, are poorly represented by the mostly inland LTAR sites. Grazinglands in the Great Plains, including the Sandhill prairies in Nebraska (NE), show low representativeness. Beyond the patch of very low representativeness in Nebraska, a swath of land from northern Montana (MT) down to southern Texas (TX) has moderate representativeness (~0.5–0.7). In California, parts of the Sonora-Mojave desert, and the California Oak Savanna region (extending into Oregon and Washington; McPherson 1997), are not well-represented due to the absence of any similar LTAR site.

**Fig. 6:**
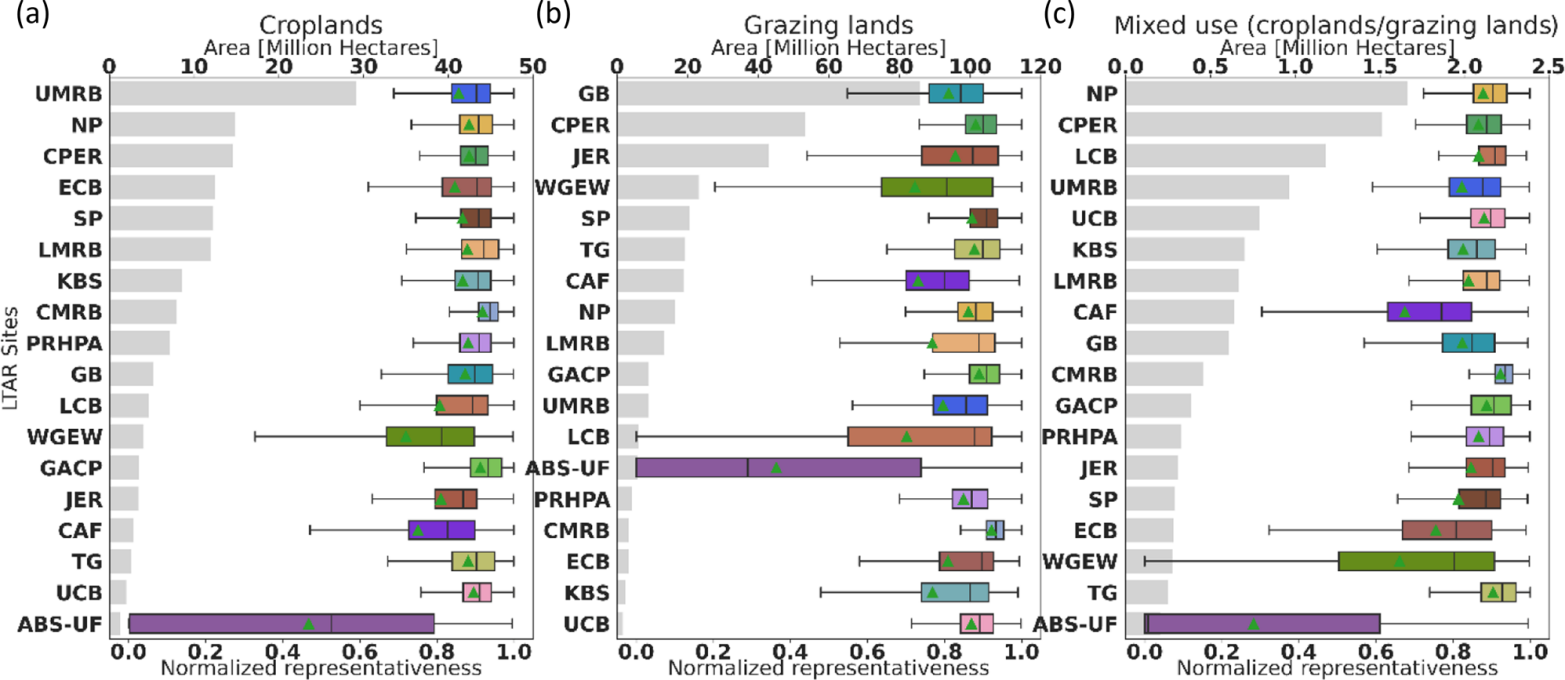
Constituency area and within-constituency distribution of representativeness by land use: **a** croplands, **b** grazinglands, and **c** mixed-use lands. Plots show the constituency area (in million hectares, top x-axis), as well as the mean and distribution of representativeness across the constituency. LTAR sites are sorted independently by constituency area (shown as gray bars) within each land use type. The colored box and whisker plots show the full range of representativeness (90th percentile as a box; mean as a bar; median as a green triangle)

Constituency areas vary widely across LTAR sites. The constituency areas range from less than 10 million hectares (Mha) up to 90 Mha, and within land use type there is large variability in how well each site represents the area within its constituency, described by its mean and distribution shown in the box and whisker plots (Fig. 6). Grazinglands have the largest constituency areas, since grazinglands are more heterogeneous, and the environmental conditions for both croplands and mixed-use working lands are more constrained. Together, the GB, CPER and JER constituencies, predominantly grazinglands, occupy the bulk of the area of working lands in the western CONUS (Fig. 6b), with CPER representativeness having the highest mean and lowest variability among them. Among croplands, the Upper Mississippi River Basin (UMRB) has the largest constituency area of approximately 29.14 Mha, with mean representativeness of 0.82 (Fig. 6a). Northern Plains (NP) represents the second largest cropland constituency representing almost 14.91 Mha, with a mean representativeness of 0.88. Ideally, an LTAR site would have a large constituency area, high mean representativeness, and low variability in representativeness across its constituency. Such characteristics would indicate that a site is well-matched for representing specific environmental conditions occurring over a large area.

### Environmental Gradients Captured by the LTAR Network

**Fig. 7:**
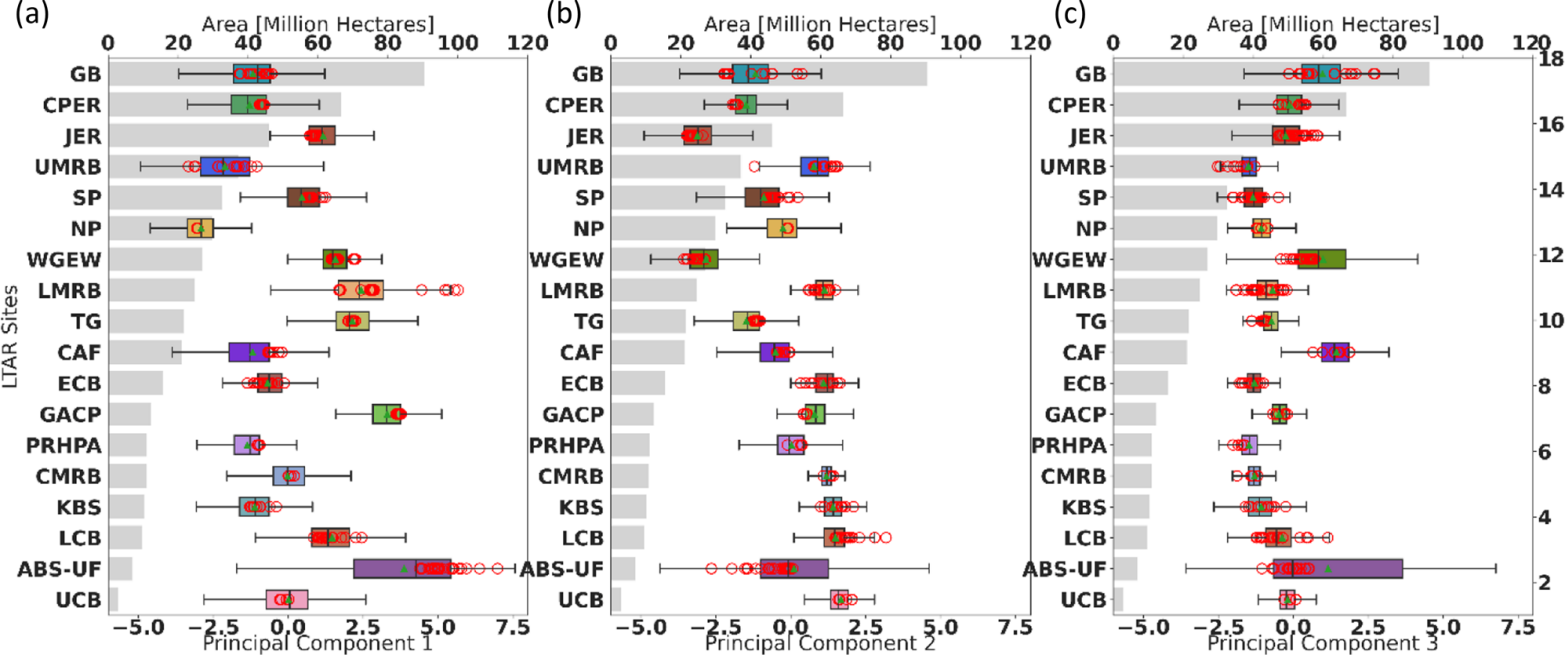
Range in environmental gradients available at each LTAR site, across the top three Principal Component Factors: **a** PC1 is interpreted as year-round warmth; **b** PC2 is year-round precipitation; **c** PC3 is the ability to store winter precipitation in the soil. LTAR sites are sorted independently by constituency area (shown as gray bars) within each principal component. Red circles show each experimental boundary centroid, mean as bar, median as green triangle, 90th percentile as box, with whiskers indicating the full range of values

Environmental gradients captured by the LTAR Network can be ascertained from the relationship between constituency areas and principal component values. Most constituency areas are well-characterized, with limited environmental variability across the constituency (Fig. 7), indicated by a narrow range of PC values. ABS-UF has the largest amount of variability for the first 3 principal components in part because its constituency area includes regions in Florida (FL) and the Pacific Northwest. Similarly, CAF has a moderate constituency area, but it is spread across a wide region, which increases environmental variability. UMRB, which has the largest constituency area (Fig. 6a), but whose area is geographically concentrated, has a large variability of the first principal component (Fig. 7a), because its constituency area includes a pronounced gradient of precipitation.

Yet constituency area is not related to environmental variability present at an LTAR site (Fig. 7). Some sites have a broad range of environmental conditions and still have limited constituency areas. For example, ABS-UF has a wide range of conditions in all the first principal components, yet its constituency is comparatively limited in area. Having a broad range of environmental conditions is not sufficient alone to cause a site to have a large constituency area.

### Leveraging Complementary Sites from Other Environmental Observation Networks

Other national-scale networks, like the NEON and LTER networks, have different programmatic criteria than LTAR, and collect different types of environmental data (Hobbie et al. 2003; Keller et al. 2008; Schimel et al. 2007). Specifically, NEON and LTER sites do not set out by design to represent working lands across the CONUS. Nevertheless, our analysis considered the possibility of a tactical “sharing” strategy of sites from different networks, to boost the representativeness of the LTAR Network. At sites within other networks identified as having environmental conditions that are poorly represented by existing LTAR sites, LTAR might persuade the sister network into adopting protocols and making LTAR measurements at those complementary sites. Ostensibly, LTAR might also adopt and perform alternative measurements at some of its identified LTAR sites in mutual reciprocity.

**Fig. 8:**
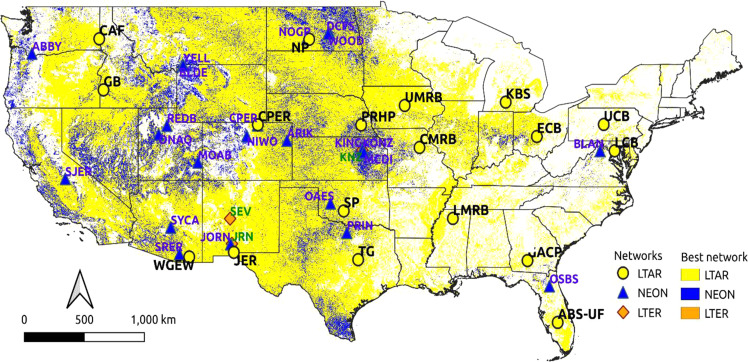
National map showing which of three national-scale networks best represents working lands across the conterminous United States (CONUS). National Ecological Observatory Network (NEON) site locations are shown in blue triangles, and Long-Term Ecological Research (LTER) sites are shown as red diamonds. The color of the areas indicates which of the three networks best represent those working lands locations: yellow is for LTAR, blue is for NEON, and green is for LTER

Only sites from the other networks that were located in working lands were considered for such complementary sharing. Figure 8 identifies some areas for which a site from the NEON or LTER network represents those CONUS locations better than any LTAR site. For such locations, the sharing strategy could be profitable, in which selected sites are identified in each partnering network to coordinate with the other, adding particular complementary data. Because this sharing has benefits to both participating networks (although the shared identified sites differ), such coordination has mutual benefits.

**Fig. 9:**
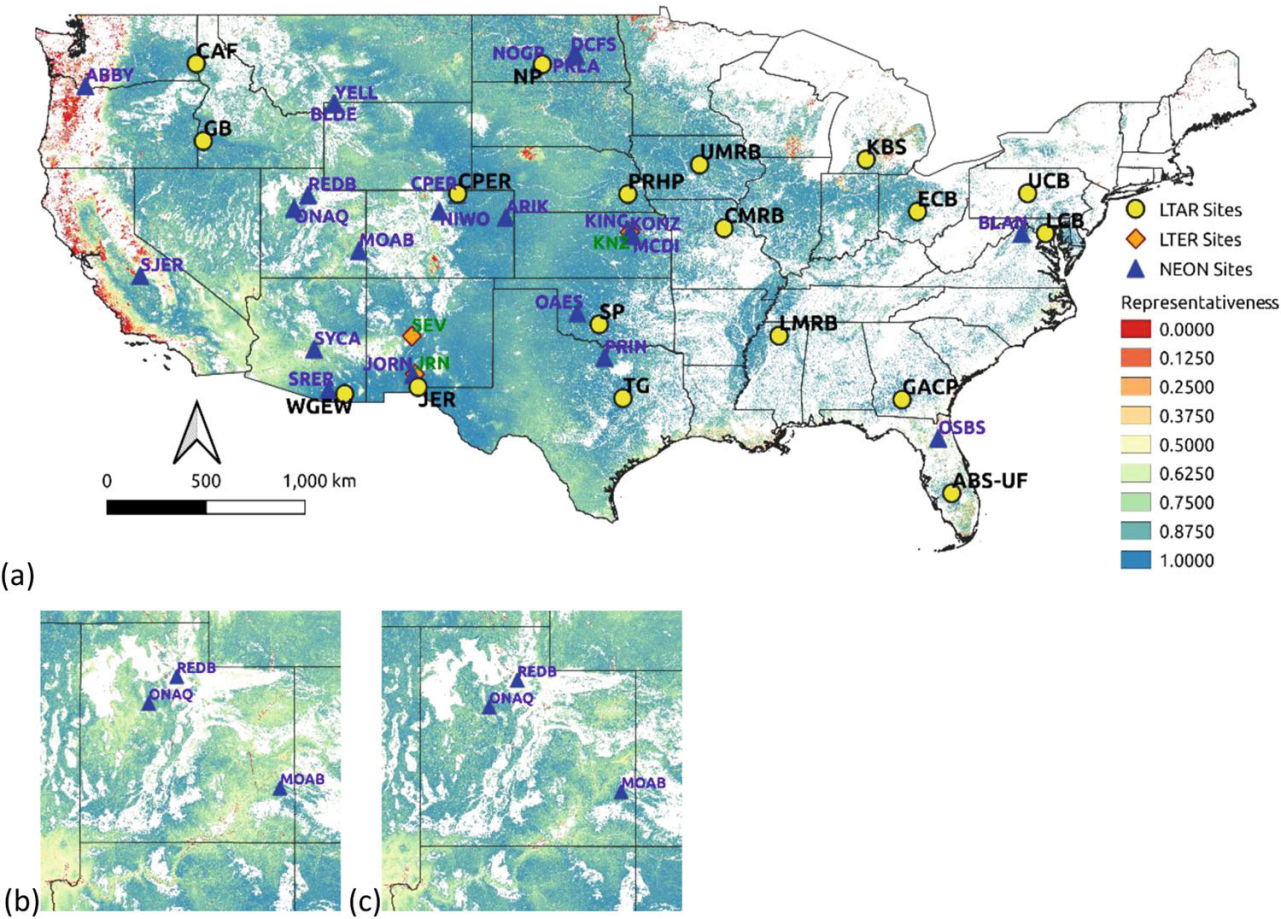
Quantifying increased LTAR representativeness of working lands by “sharing” sites from two other national-scale networks, NEON and LTER. In the national map (**a**), LTAR site locations shown as black dots, NEON sites shown as blue triangles, and LTER sites shown as red diamonds. Insets show before (**b**) and after (**c**) the addition of NEON and LTER sites to LTAR representativeness in Utah

**Fig. 10:**
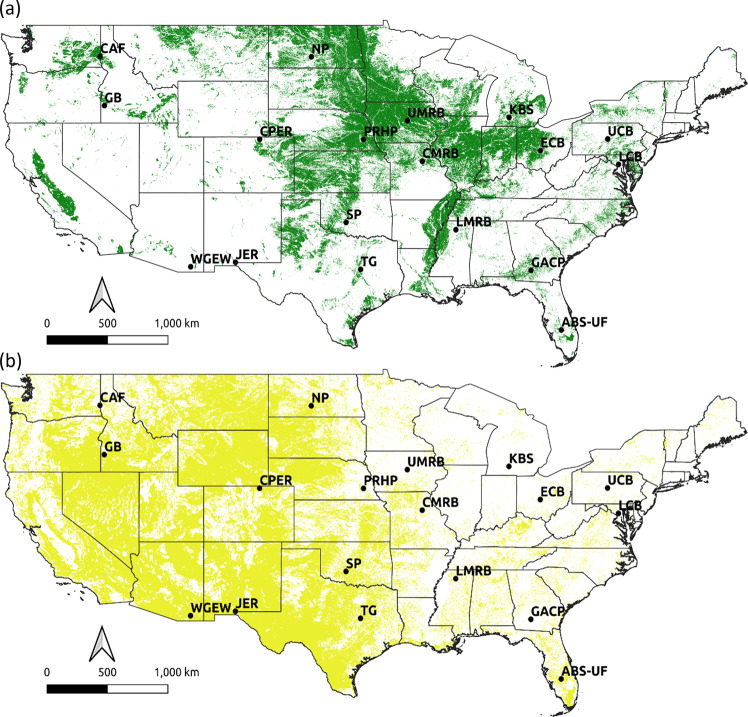
Maps of “mask” datasets used to limit the analysis: **a** croplands mask; **b** grazinglands mask (unpublished data)

**Fig. 12:**
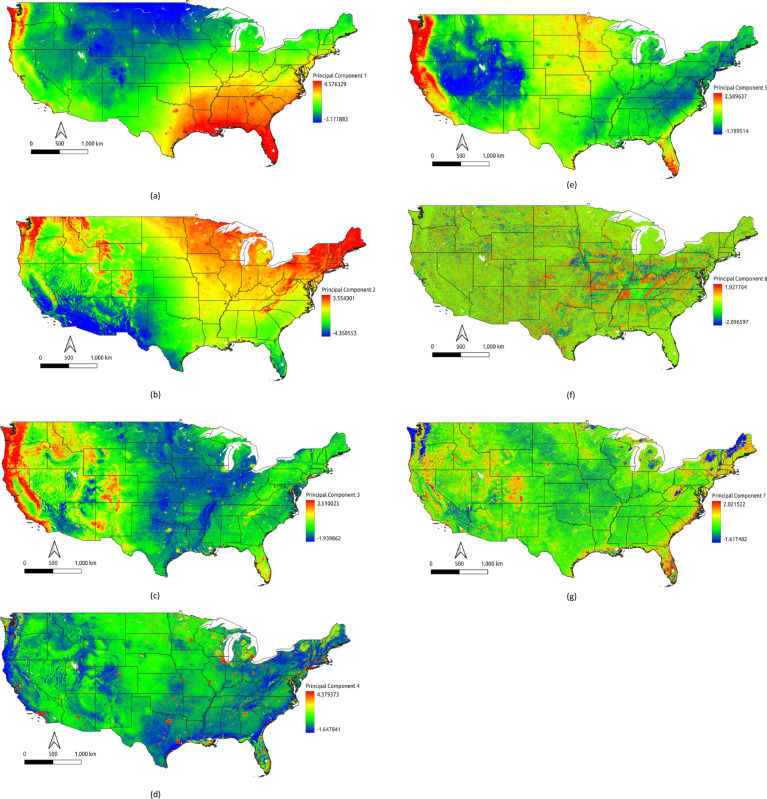
Spatial representation of top seven principal components used in the analysis (**a**–**g**). Components representing various data dimensions show range of spatial variability and patterns across continental United States. **a** Principal Component 1 explains 28% of variance in data and is interpreted as year-round warmth. Eastern and southeastern urban areas show up particularly well. **b** Principal Component 2 explains 20% of variance, and is interpreted as year-round precipitation where values are high. Rain in the pacific northwest and snow in the northeast show well, as do the southern regions where seasonal precipitation patterns are affected by monsoonal and subtropical climatic patterns. **c** Principal Component 3 explains 13% of variance and is interpreted as the ability to store winter precipitation in the soil. **d** Principal Component 4 explains 9% of variance and is interpreted as describing the consolidation and texture of soils. Urban areas show as being highly compacted (high bulk density) and low in fertility. **e** Principal Component 5 explains 6% of variance and loads primarily on soil water holding capacity. **f** Principal Component 6 explains 6% of variance and loads primarily on soil nutrients, precipitation seasonality, and topographic index. **g** Principal Component 7 explains 5% of variance and loads primarily on precipitation seasonality, snow, and soil nitrogen without carbon

Twenty-eight NEON sites (Table 3) thus located were considered for sharing in this analysis. Of those, sites located in the Great Plains, intermountain West and Pacific Northwest, increased the representativeness of the LTAR Network, denoted by the blue pixels in Fig. 8 and overall increases in representativeness in those areas (Fig. 9). Of the three LTER sites located in working lands, the Sevilleta LTER site in southern New Mexico is the only LTER site that improves LTAR representativeness. The LTAR Network was designed to represent working lands, which, according to our analysis, it does. Because NEON and LTER were not designed with this objective, a network level comparison of working-lands representativeness is spurious and not commensurate with their purposes. Nevertheless, the map in Fig. 8 shows some locations in blue (and a few in green) where some sites in these networks unintentionally provide greater representation of working lands than LTAR. Benefits to LTAR representativeness from sharing NEON sites are potentially greater than from sharing LTER sites, and disproportionately accrue to grazing- and mixed-use lands.

## Discussion

For this analysis, the selected method evaluated the representativeness and constituency of the LTAR Network as it exists, providing a result that is immediately useful for scientists and managers to evaluate the similarity of their own sites to other areas. The pairwise Euclidean distance was measured between each LTAR experimental boundary polygon and each 1 km grid cell of the CONUS, across 7 dimensions of environmental principal components. This method best examines the representativeness of an existing network and its set of sampling locations, as opposed to designing a network.

Results describing the variability of principal component values indicate that the environments for each of the 18 LTAR are well-differentiated (Fig. 3a), and the unique environmental conditions across the CONUS are sampled well. As a whole, the LTAR Network representativeness of environmental conditions across the CONUS was high, but increased even more after application of masks delimiting “working lands” of cropping and grazing systems. This analysis uses climatic and edaphic conditions selected to reflect primary environmental drivers for agricultural productivity, rather than agricultural outputs themselves. Thus, our analysis represents potential, rather than currently realized productivity.

Constituencies of the 18 LTAR sites resemble ecoregions to which they are related. Unlike ecoregions, however, each constituency region is defined by environmental conditions at a particular LTAR site. Most constituencies are spatially contiguous (except where interrupted by the working lands mask), with a few notable exceptions. The dark purple R.J. Cook Agronomy Farm (CAF) constituency forms a high-elevation ring around the area excluded from the cyan Great Basin (GB) and intermountain west constituency. Most notably, the light purple Archbold Biological Station – University of Florida (ABS-UF) constituency is split across sides of the CONUS. This Florida LTAR site is currently the best representative LTAR site for locations in the California Oak Savanna area of the Pacific Northwest. This result may be surprising until noting that LTAR representativeness is lowest in these same Pacific Northwest locations (Fig. 5a), revealing that, although ABS-UF is the current best LTAR site representative among all LTAR sites, its representativeness of these areas is still poor.

Constituencies shown in Fig. 5b will make intuitive sense to most ecologists. These areas resemble ecoregions but differ from normal ecoregions by having their environmental conditions “centered” on those at particular existing LTAR sites. While intuitive, it would have been difficult to predict such a constituency map before having done this analysis. Comparing Fig. 5a, b with the areas shown in Fig. 1, which were the areas of best representation presumed by the LTAR site scientists, provides an indication of the difference between preconceptions and quantitative analyses. On the other hand, our analysis did not consider key features of LTAR site research programs, such as the empirical understanding of dominant regional production systems and practices related to crop and livestock management.

Representativeness and constituencies computed here only reflect similarities in the primary environmental and edaphic conditions. In most cases, agricultural productivity will be correlated with primary growing conditions, and it would be useful to characterize network representativeness and constituency on that basis. Other important differences in agronomic practices, or other social, economic or political treatments were not considered in this analysis, and may dramatically affect, alter or override the similarities in primary environmental conditions shown here. Hence, the delineation of constituency areas would likely change if additional datasets describing a more holistic approach to socio-agroecosystems were integrated into the analysis. For example, while the CAF constituency shown here forms a ring-like feature, the LTAR research at CAF is focused on cropland production and associated agronomic practices common throughout the region shown in Fig. 1, a fact that was noted in the reasoning provided for the initial CAF boundary delineation (Bean et al. 2021). In contrast, research at the GB is oriented toward a different set of agronomic practices focused on grazingland production. Therefore, if such practices were included in our analysis, the actual CAF constituency may resemble more strongly the areas shown in Fig. 1.

Although our goal was not the design of a network de novo, but instead the analysis of the existing LTAR network of sites, it identifies poorly represented regions and provides guidelines for network growth. In Fig. 6, we can distinguish LTAR sites with large constituencies and high mean representativeness with low variance across them. The combination of environmental conditions at these sites tracks very well with the environments in many locations across the CONUS so that these LTAR sites have generalist conditions best-representing expansive areas. In Fig. 6b, the top six LTAR sites in the grazinglands panel are such “generalist” sites. But other LTAR sites can be identified which also have high mean and a narrow range of representativeness with smaller constituency areas. Despite having environments that are just as well-suited to their constituency locations, these LTAR sites are specialized environments, and these “specialists” are the best LTAR representatives for smaller, more focused (and geographically limited) areas. GACP, UCB, and CMRB are examples of such specialist LTAR sites for grazinglands (Fig. 6, left). LTAR sites with a large constituency have generalist environments, while those with smaller constituency areas have more specialized environmental combinations.

Generalist sites are not necessarily better to have in a network than specialist sites, despite the fact that they enjoy greater constituency area. Unusual or unique areas are represented better by specialist sites, and networks need such specialist sites to capture the more unique environments that they hope to represent. Young networks having few sites will increase their overall representativeness fastest by initially adding generalist sites having average environments. But, as the network grows, a switch to accruing specialist sites that are targeted to represent unique environments, increases the depth of representativeness in the network. This strategy to add generalist sites first, and then targeted specialist sites, will serve both new and existing networks as they grow. Identifying the best targeted specialists for addition is increasingly difficult without strategic guidance like that provided by the quantitative statistical network analysis provided here.

A moderate mean and a wider range with a lower bottom end representativeness may indicate a generalist site that is being forced to represent some fairly distinct environments rather poorly. The lower representativeness in these secondary locations decreases the mean representativeness and increases the representativeness range. While this site is still the best, it is a forced, poor fit in these secondary locations. Existence of such sites would indicate a strong need for an additional new site to be added to the network to better represent the unusual environments in this unique secondary area. These forced “double duty” characteristics are shown in WGEW and CAF, as well as LCB in grazinglands, (Fig. 6), and they may be acting as poor representatives for portions of their constituencies.

The ABS-UF site has a small constituency that is split across the width of the CONUS. This site also shows one of the widest ranges of representativeness across its constituency. In addition to signifying the low (but still best) representativeness values that ABS-UF has in the Pacific Northwest, the site itself contains environmental heterogeneity across its 33 large experimental boundary polygons. Two distinct environments comprise ABS-UF: Buck Island Ranch, in the center of the Florida peninsula, draining into Lake Okeechobee, and the UF Research Station near Ona, closer to the Gulf of Mexico coast. This variety of environmental conditions at this site contribute to its ability to act as a poorer surrogate for otherwise un-represented CONUS locations, as well as contributing directly to broadening the range of representativeness for its constituency. If additional LTAR sites were added in these poorly represented areas, it is likely that the range of representativeness for ABS-UF would then become more defined.

While the LTAR Network is now in its second decade of existence, the careful addition of targeted, unique specialist sites would support a strategic approach to increasing representativeness. One of the best ways to identify potential locations for these additional needed specialist sites is to consider the minima shown in the representativeness map, like the Pacific Northwest or coastal California (Fig. 5a). Such locations represent theoretical optima, but pragmatic and infrastructural concerns may preclude the establishment of new sites at exactly the suggested minimum representativeness locations. In these cases, nearby compromise locations could provide nearly as much targeted increase in network representativeness.

Sources of uncertainty in results were related to the resolution of the input datasets, which were harmonized to a resolution of the coarsest datasets (1 km climate). Data were available for some variables at higher resolutions, e.g., land cover masks, and the LTAR experimental boundary polygons were mapped at much finer scales. However, the LTAR polygons were treated even-handedly, with mean environmental conditions at the polygon centroids used as the value for those locations, regardless of actual polygon size. Hence some variability was potentially unaccounted for during this resampling process. Likewise, interpretations of these results are constrained to the minimum mapping unit of the output datasets, and should only be interpolated to finer resolutions with an additional downscaling step, which was not done here. For CONUS, the 1 km grid provided the highest resolution possible using best-available “state-of-the-art” data.

## Conclusions

These analyses were exhaustive and compared every 1 km map cell location with every other across the CONUS. We performed no generalizations or classifications from clustering locations with similar conditions together; but rather, exact multivariate similarities/differences were calculated between all possible locations in the CONUS. Any errors and uncertainties inherent in the 15 environmental data layers were inherited by our analysis. Nevertheless, for its 18 sites, the LTAR Network has good representativeness of environmental conditions that drive productivity across the CONUS.

These exhaustive comparisons also permit the study of within-LTAR site representativeness, through the use of separate representativeness maps relating to the centroids of experimental areas within each LTAR site. For example, a researcher interested in replicating an experiment in a similar landscape, could use this more detailed information to evaluate comparable sites within a particular LTAR region. Similarly, an LTAR site might be guided by their own within-site representativeness to differentially develop and enhance experimental support infrastructure for field research sites in those places that have the greatest potential for enhanced national representativeness, thereby increasing impact and service to LTAR stakeholders.

Constituency of LTAR sites can be used to prioritize the establishment of experimental research at or even within particular sites, or to identify which sets of experimental boundaries should be considered when generalizing knowledge at any point in the CONUS. The constituency map shown in Fig. 5b is based on the single LTAR site having the greatest representativeness at that location. While that single site is the best, most-representative LTAR site, the 2^nd^, 3^rd^ and subsequent level constituency sites may also have good representation for that location. Although not shown here, a researcher could use these lower-order constituencies at any set of locations to generate a customized multiple site constellation of LTAR locations at which to perform a field experiment designed to maximize representativeness over a region of the CONUS, taking advantage of all replication possible within the existing LTAR network.

Improvement of representativeness could be obtained through site-sharing collaborations with other research networks such as NEON and LTER. Most of the improvements brought by NEON sites are in the central and western working lands, including the land overlaying the Ogallala aquifer, which was poorly represented by LTAR. However, while representativeness is improved for California, Oregon, and Washington working lands, the representativeness remains poor for a large fraction of those areas. The US west coast is an overlapping intersection of many environmental gradients, making representation of these areas difficult to capture.

Unless different national networks measure the same variables, benefits resulting from their combination remain theoretical, and it is unrealistic to expect networks designed for different monitoring purposes to measure non-target variables. It may be practicable, however, for networks to take on measurement of certain complementary variables at one or a few most-complementary sites, and, through mutual cooperation, for both networks to realize boosts in their own national representativeness. Cross-network representativeness analyses like this one can identify, direct, and limit the complementary sites for such beneficial additional measurements, making them achievable and mutually motivated. Adding measurements at an existing site in a sibling network may be more effective than establishing and maintaining a new site in the home network, and will increase the efficiency of limited funding resources.

As a collection of geographic locations where ecological samples or measurements are taken, it is a common requirement for ecological networks to scale measurements up to broader regions in statistically valid and meaningful ways. Representativeness and constituency analyses can be performed on any network, *sensu lato*. Whether an official permanent network with considerable developed infrastructure and resources at each site, or just a set of locations where samples have been taken, any network is amenable to this quantitative evaluation. Nor does the spatial extent of the network affect the applicability of representativeness analysis; whether comprising a single state (Hoffman et al. 2013), a nation or continent (Sundareshwar et al. 2007), or a global extent (Kumar et al. 2016; White et al. 2005). Indeed, global networks like Fluxnet were created by combining many different individual national-scale flux networks (Kumar et al. 2016).

While our LTAR Network representativeness analysis exhaustively considered principal environmental drivers related to production on working lands, we did not differentiate among LTAR sites with respect to the focal agronomic systems under study, nor the socio-economic context within which those agronomic systems are embedded. Environmental similarities, representativeness, and constituencies form a basic and fundamental first-order understanding of the production potential of agronomic systems across the CONUS. Future analyses that combine relevant features of human dimensions, with realized agricultural production outputs and environmental characteristics, will provide further insights into the LTAR Network’s representativeness of these national socio-agroecosystems across the United States.

## Figures and Tables

**Fig. 11:**
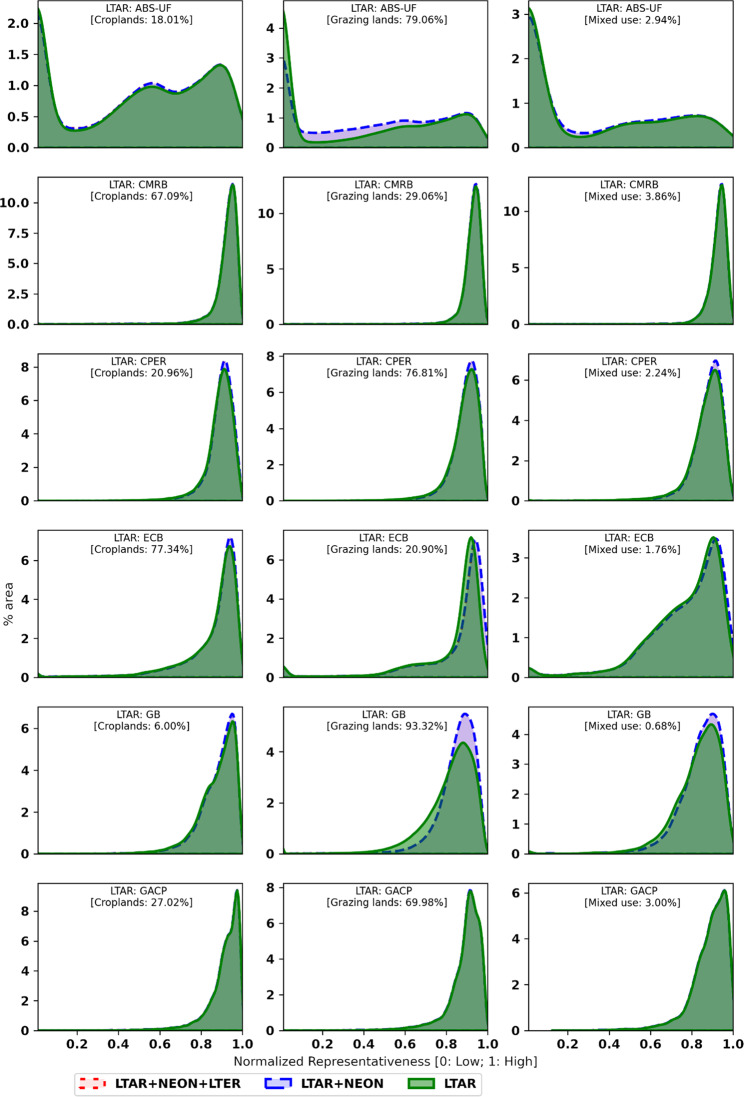

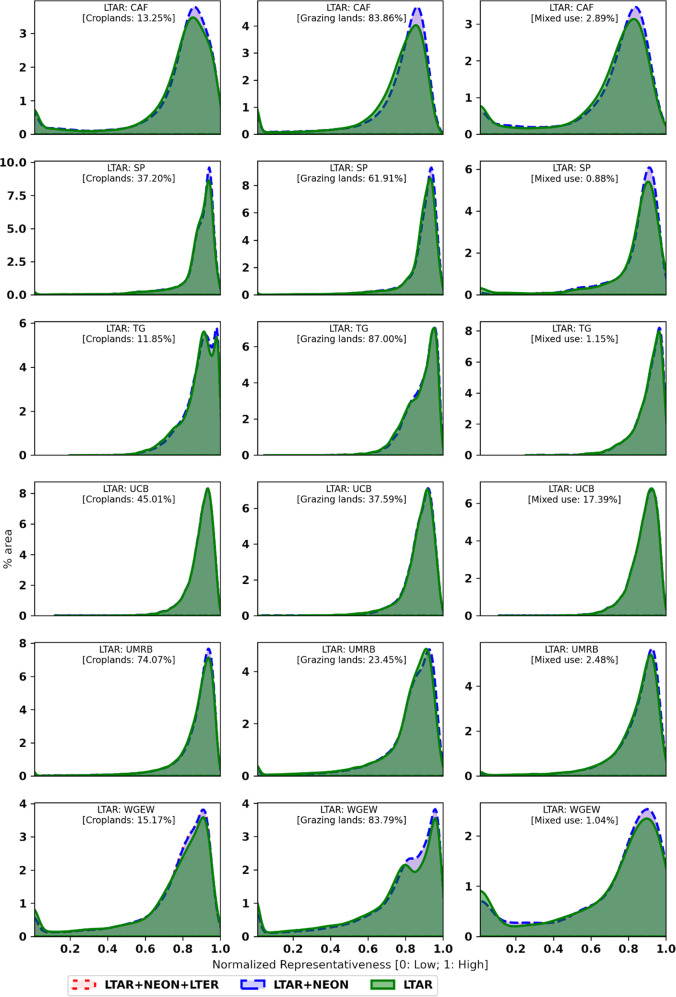

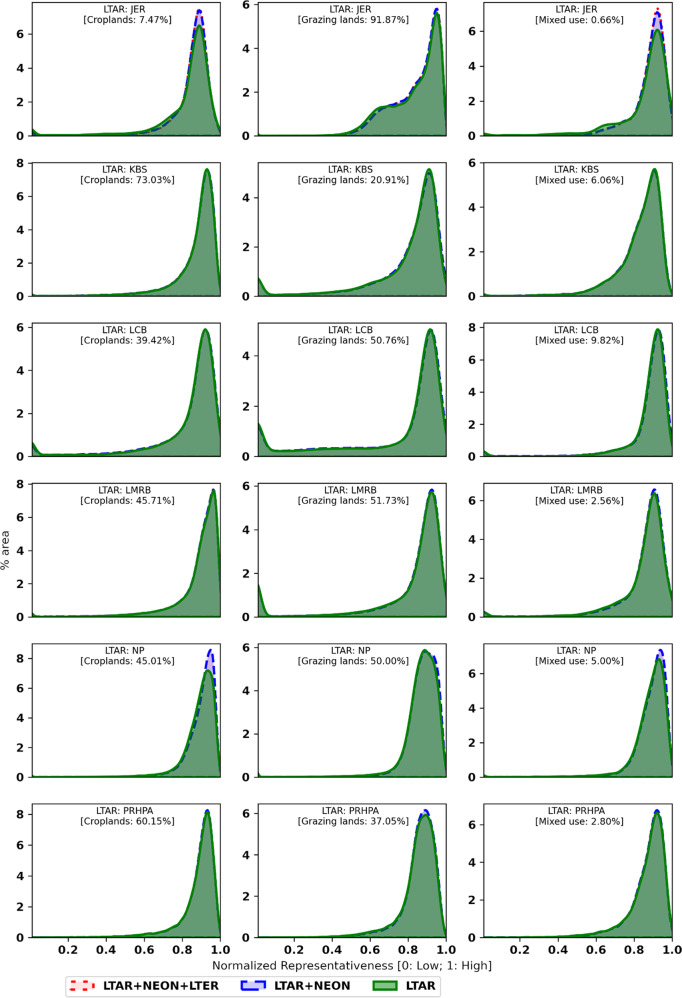
Distribution of normalized representativeness within each LTAR site constituency for different network scenarios. While addition of LTER sites does not improve representativeness aside from a small region in JER, NEON sites improve the representation of the LTAR Network significantly in a number of LTAR site constituencies

## Data Availability

All data produced by this research are available in the following repository at Zenodo.org: 10.5281/zenodo.7106385. All code used for analyses is available in a repository at Github.org, also linked through the Zenodo project link: 10.5281/zenodo.7539722.

## Appendix

Figures 10–12; Tables 2–4

**Table 2:** LTAR site names with number of experimental plots or fields

#	Site ID	Site Name	Number of experimental boundary centroids
1	ABS-UF	Archbold -University of Florida	33
2	CAF	R.J. Cook Agronomy Farm	349
3	CMRB	Central Mississippi River Basin	8
4	CPER	Central Plains Experimental Range	25
5	ECB	Eastern Corn Belt	21
6	GACP	Gulf Atlantic Coastal Plain	27
7	GB	Great Basin	28
8	JER	Jornada Experimental Range	36
9	KBS	Kellogg Biological Station	29
10	LCB	Lower Chesapeake Bay	162
11	LMRB	Lower Mississippi River Basin	119
12	NP	Northern Plains	70
13	PRHPA	Platte River High Plains Aquifer	6
14	SP	Southern Plains	59
15	TG	Texas Gulf	194
16	UCB	Upper Chesapeake Bay	242
17	UMRB	Upper Mississippi River Basin	15
18	WGEW	Walnut Gulch Experimental Watershed	106

**Table 3:** NEON site names

#	Site ID	Site Name
1	ABBY	Abby Road NEON
2	ARIK	Arikaree River NEON
3	BLAN	Blandy Experimental Farm NEON
4	BLDE	Blacktail Deer Creek NEON
5	CPER	Central Plains Experimental Range NEON
6	DCFS	Dakota Coteau Field Site NEON
7	JORN	Jornada Experimental Range NEON
8	KING	Kings Creek NEON
9	KONA	Konza Prairie Agroecosystem NEON
10	KONZ	Konza Prairie Biological Station NEON
11	LEWI	Lewis Run NEON
12	MCDI	McDiffett Creek NEON
13	MOAB	Moab NEON
14	NIWO	Niwot Ridge NEON
15	NOGP	Northern Great Plains Research Laboratory NEON
16	OAES	Marvin Klemme Range Research Station NEON
17	ONAQ	Onaqui NEON
18	OSBS	Ordway-Swisher Biological Station NEON
19	PRIN	Pringle Creek NEON
20	PRLA	Prairie Lake NEON
21	PRPO	Prairie Pothole NEON
22	REDB	Red Butte Creek NEON
23	SJER	San Joaquin Experimental Range NEON
24	SRER	Santa Rita Experimental Range NEON
25	STER	North Sterling NEON
26	SYCA	Sycamore Creek NEON
27	WOOD	Chase Lake National Wildlife Refuge NEON
28	YELL	Yellowstone National Park NEON

**Table 4:** LTER site names

#	Site ID	Site Name
1	JRN	Jornada Basin LTER
2	KNZ	Konza Prairie LTER
3	SEV	Sevilleta LTER
